# The Bleaching of Discolored Teeth

**Published:** 1898-01

**Authors:** J. E. Nyman

**Affiliations:** Chicago.


ARTICLE VII.

### THE BLEACHING OF DISCOLORED TEETH.

BY J. E. NYMAN, D. D. S., CHICAGO.

Read before the Chicago Dental Society, June 1, '97.

In the consideration of this subject it may be well to consider first the causes of discoloration. In most of the cases that come to us the teeth have putrescent pulps in them, or it is an abscessed tooth that has been treated and the root canal has not been properly filled. One product of all decomposition of organic tissue is sulphuretted hydrogen, and that coming in contact with the oxyhæmoglobin of the blood forms a sulpho-methæmoglobin which is of reddish green tinge. A reddish green usually expresses the tinge of discoloration we find, because a reddish green is very much like a dirty brown. A strange occurrence which some of you may have experienced, is that if a patient happens to appear to you on one of your busiest days with a tooth that shows unmistakable signs of an abscessed condition, and you are able only to take the time to relieve that patient of the pain by drilling into the canal, and allowing it to discharge its putrescent contents, telling the patient to come in again in a day or two or three, as the case may be, you may be surprised when that patient returns on that second or third day to find that the tooth is discolored worse than it was when you first saw it. There is a reason for that change for the worse, and it is this : If during the action of the sulphuretted hydrogen on the oxyhæmoglobin of the blood oxygen be present the reaction is much more marked and the color formation much more decided, therefore in the case we have just remarked upon, in making a vent for the abscess we have also established an air passage by means of which the air containing oxygen has intensified the reaction of the sulphuretted hydrogen on the oxyhæmoglobin of the blood, causing more decided discoloration.

The causes of discoloration may be divided into two classes, organic and inorganic. One of the organic causes is that which I have just mentioned, namely, sulpho-methæmoglobin; another the oil of cinnamon when excessively used. These seem to comprise the organic causes. Now, strictly speaking, the causes that I am now about to give are not entirely inorganic, because organic chemistry has played some part in the chemical reactions of these, so to speak, inorganic causes. We will first consider iron.

Miller says that iron is the most widely distributed of all the metals in the human system, and it plays the most important part in the physiology of life, that he finds it present in the vascular tissues, in the pulp and in the pericementum. Now, then, if you come across one of those cases where there is little more iron than is normal in the blood and an abscessed condition has been established in addition to the discoloration from the sulpho-methæmoglobin, there will be a discoloration due to the sulphide of iron. Again, should the patient be suffering at the time this abscessed condition takes place from ptyalism, there would likely be present another discolorizing combination—sulphide of mercury. Then, again, there may be a little excess of manganese in the blood. In that case there might also be formed a sulphide of manganese. There may also be discoloration due to the sulphides of tin and silver, owing to the action of sulphuretted hydrogen arising, it may be from the decomposition of debris of food upon remnants of old amalgam fillings. Another cause of discoloration that I might also mention is that which may be due to the action of the medicaments used, upon the steel in the instruments used; and I believe Dr. Harlan has stated that there may be a discoloration resulting from the use of carbolic acid in the treatment of devitalized teeth. All of these sulphur combinations are of a dark color, varying from brown to black.

These complications tend to make a bad matter worse, although I frankly admit that it is almost impossible to tell just which of these causes has been most actively at work in any one case of discoloration, but it is highly probable that in the majority of cases that come to our notice, it is mainly due to the sulpho-methæmoglobin.

Now, just a word as to what bleaching is. The best definition that I can find is the one given by Dr. E. C. Kirk, in an article in the *Cosmos*, in which he defines it as "the reaction between a substance having color and some substance capable of so affecting the integrity of the molecule of the coloring matter as to cause it to lose its distinguishing characteristic, its color." There are two classes of bleachers, namely, the oxidizers and the reducers. The oxidizers are those which act by oxidizing the coloring material or extracting the hydrogen from it. The reducers acting in exactly the reverse; abstracting the oxygen from the coloring compound. As examples of oxidizers we may mention peroxide of hydrogen, potassium permanganate, chlorine and the chlorine compounds, and as reducers sulphur dioxide and oxalic acid. The ones most universally used are the chlorine compounds and the peroxide of hydrogen, although some use is made of oxalic acid.

Now, necessarily there must be some choice of bleachers, and the question is, which shall it be? Unfortunately for the oxalic acid process we are quite divided as to the exact action of oxalic acid on the structure of the teeth, as to whether it does or does not injure it. However, as I said before, the bleaching agents most used . are the chlorine compounds and the peroxide of hydrogen solutions. The objections to chlorine and its compounds are that chlorine has a disagreeable odor, that it affects all steel instruments, that it is such an irritant to the tissues, to say nothing of the possible formation under some circumstances of hydrochloric acid. Personally I do not see how this can take place, but I have heard it mentioned as something that might happen with the use of the chlorine process. This leaves us only the peroxide of hydrogen solutions to consider, and they really seem to be the best of all, because oxygen is a bleacher of world-wide reputation and the decomposition of peroxide of hydrogen gives us nothing but oxygen and a residuum which is nothing more or less than water.

I might, in passing, speak of a very peculiar case which Dr. Sanger, of New Jersey, reported, in which, after successfully bleaching a tooth by the chlorine method, the tooth rediscolored. The only cause he could discover was that a proximal gold filling projected into the enlargement he had made in the pulp cavity. Then he made up his mind that this rediscoloration was caused by the chloride of gold. He said he removed the filling and rebleached the tooth, after taking precaution to wash out the chloride of gold stain, and then the tooth did not discolor again. It does not seem possible to me that this could have been the cause, because free chlorine will not affect gold. There must be a combination of the two acids, hydrochloric and nitric, popularly known as aqua regia, before chlorine will affect gold. I am inclined to think that that tooth needed another bleaching, and that it was not done thoroughly the first time.

Now, as to the method of procedure when a patient presents himself with a discolored tooth which he wishes bleached. In the first place, do not promise him too much for there are cases that cannot be bleached, at least cannot be bleached successfully. In the second place, after treatment and curing of the abscess, if there be one, fill the root canal well with gutta-percha or other material that will hermetically seal the apical end. Then enlarge the canal as you would to insert a post for a crown. Thoroughly cut out all the discolored dentine that you can without materially weakening the tooth, because in doing that you very effectually remove part of the discoloration, and leave less work for your bleaching agent to do. Now, the question presents itself as to whether we shall make use of our recent knowledge and appliances in editorial matters, or simply adopt the old time method of bleaching teeth. I prefer the modern method, as it is evident that the action of the electric current makes a freer and more abundant formation of oxygen that can be obtained under ordinary methods, and Dr. Morton's experiments seem to prove that the cataphoric action causes the oxygen to penetrate deeper than it would by ordinary osmosis. But I am very much in doubt whether to call the electrical process cataphoresis or electrolysis. Looking at it from some points of view it seems as if it were electrolysis. Looked at in the light of the experiments of Dr. Morton, of New York, it looks as if cataphoresis were the term for it. A very curious thing about which I am as much in the dark as is any one else is as to just how the oxygen does the bleaching under the action of the electric current. The oxygen has an affinity for the positive electrode and will collect there, while the hydrogen will be driven to the negative electrode. It may do the bleaching by expelling all the hydrogen from that discolored compound, and driving that into the tissues toward tho negative electrode, allowing it to be replaced with the oxygen, or it may be that the bleaching takes place owing to the fact of a constant accumulation of oxygen in the region of the positive electrode, giving such an excess that the tissues of the tooth take up some part of it. Which of these two theories is correct I do not know. There have been some appliances devised for bleaching the teeth cataphoretically, so to speak by Dr. Hollingsworth. They consist, first, of a rubber nipple, which is expanded by means of a nipple expander. After the rubber dam has been adjusted and the preliminary preparation of the teeth completed, this nipple is slipped over the tooth to the neck of it and the expander is withdrawn, leaving the tooth covered with the nipple. A little glass tube with a spiral platinum wire in it is then inserted into the nipple, closing over it and holding it fast. There are also two little hooks attached that are hooked over the fold of the rubber dam as it is stretched across the patient's face, and support the little tube. Take a tube of 25 per cent. pyrozone, which should contain two drachms of pyrozone, wrap it in a towel or napkin which has been soaked in ice water, open it by cutting off the tip with a diamond disk, and empty the contents into an ordinary glass tumbler. 25 per cent. pyrozone is an ethereal solution and an ethereal solution is a very poor conductor of an electric current. So add to this ethereal solution of pyrozone an equal amount of warm water, to which has first been added a grain of sulphide of zinc to increase the conductivity of this solution. The ether should then be evaporated by gentle heat until you no longer get fumes of it from the solution. The solution will then be aqueous instead of ethereal and will be ready for use. The stopper is now removed from the little glass tube. A double bulb syringe which I will describe has been devised for filling it. The further or second rubber bulb of this syringe is first collapsed and the point of the syringe inserted in the pyrozone solution and this second bulb is then allowed to fill with the pyrozone solution. A little platinum tube runs clear through this outer rubber tube into the second bulb; but the second bulb has no connection whatever with the first bulb. Attached to this first bulb is a rubber tube which envelopes the platinum tube and which projects down almost to the end of it; between these two tubes there is a space. Now, this first bulb is collapsed, the rubber point of the syringe is inserted in this little glass tube and the bulb released. The moment that the first bulb is released the air is drawn out of the glass tube into it, creating a suction which causes the space in the tube to be filled with the pyrozone solution from the bulb. If you attempted to force the pyrozone solution into the tube in the ordinary manner, the air pressure would be so great that you would spill most of it and put but a small quantity of it in the tube. The positive electrode which has been devised for this appliance has a clamp which fits around the neck of this glass tube, and comes in contact with the platinum coil of wire. When this has been attached the open end of the tube is closed by means of a stopper. Great care should be taken that the joint between the neck of the tooth and the rubber dam is hermetic; because if it is not the current will be leaking out around the mouth. The negative electrode is attached to the wrist. Now, when all these precautions and these various steps have been taken the current can be turned on very rapidly, because there is not the sensitiveness to contend with that there is in a live tooth. It is said that a current of the strength of twenty-five volts for half an hour will on an average do the work. I am very free to admit that the majority of cases in which I have tried this process I have not bleached to my own satisfaction in that time. I have done it mainly at clinics, where I do not have the opportunity of seeing the cases again. But I know that Dr. Stewart, of this city, succeeded in bleaching a tooth perfectly in that time by this method at one of the clinics. I have had some cases, however, that have been a success. It is said that if a milliampere meter records more than three-quarters of a milliampere before a twenty five volt pressure is reached it indicates that there is a leak somewhere, so great precaution should be taken about the joint at the neck of the tooth. After bleaching the cavity the tooth should be hermetically closed by cement and gold.

There is one objection to this appliance, and that is that it hides the tooth from view while the bleaching process is going on. But in point of fact, many bleaching operations have to be repeated two or three times before fully satisfactory results are obtained. However, it is claimed that the full action of the pyrozone is not evident until twenty-hours have elapsed, so that when the patient returns later the tooth may be found perfectly bleached, although when you dismissed the case it may have seemed to you to have been a failure. This thought has occurred to me: that probably not all of the cells in a discolored tooth are discolored at one time, and by bleaching out those that have been discolored the tooth regains its normal color on account of the large majority of cells which were not affected by the discoloring agent. If it were a fact that all the cells were discolored at one time, I do not believe that you would ever be able to restore the normal color to such a tooth. Consequently you will have some cases that will be failures in spite of all that you can do with them, due probably to the condition I have just spoken of, namely, that all of the cells of the tooth were affected by this discoloring agent. And even if you succeeded in bleaching such a tooth you would have as unnatural a color as it was before you bleached it. There are some cases in which a rediscoloration takes place owing to the fact that there are cracks in the enamel which allow the absorption of discolorizing agents from the mouth. I do not understand organic chemistry well enough to give you the chemical composition of the various discolorizing agents, or to be able to write out the chemical reaction which takes place between the discolored material and the bleaching fluid; in fact, there are many things about this subject that I would like to explain to you, but I really do not understand them myself.—*Dental Review.*

